# The effect of lip closure on palatal growth in patients with unilateral clefts

**DOI:** 10.7717/peerj.9631

**Published:** 2020-07-30

**Authors:** Robin Bruggink, Frank Baan, Gem Kramer, Colet Claessens, Anne Marie Kuijpers-Jagtman, Ewald M. Bronkhorst, Thomas J.J. Maal, Edwin Ongkosuwito

**Affiliations:** 1 Department of Dentistry - Orthodontics and Craniofacial Biology, Radboud University Medical Center, Nijmegen, The Netherlands; 2 Radboudumc 3DLab, Radboud University Medical Center, Nijmegen, The Netherlands; 3 Radboud Institute for Health Sciences, Radboud University Medical Center, Nijmegen, The Netherlands; 4 “Alkmaarse Orthodontisten”, Noordwest Ziekenhuisgroep, Alkmaar, The Netherlands; 5 Department of Orthodontics, University Medical Center Groningen, Groningen, The Netherlands; 6 Department of Orthodontics and Dentofacial Orthopedics, University of Bern, Bern, Switzerland; 7 Faculity of Dentistry, Universitas Indonesia, Jakarta, Indonesia; 8 Department of Dentistry - Preventive and Restorative Dentistry, Radboud University Medical Center, Nijmegen, The Netherlands; 9 Department of Oral and Maxillofacial Surgery, Radboud University Medical Center, Nijmegen, The Netherlands; 10 Amalia Cleft and Craniofacial centre, Radboud University Medical Center, Nijmegen, The Netherlands

**Keywords:** Orthodontics, Dental models, Imaging, Three-Dimensional, Cleft palate, Diagnostic imaging, Maxillofacial development

## Abstract

**Objectives:**

The objective of this study was to compare maxillary dimensions and growth in newborns with Complete Unilateral Cleft Lip and Palate (UCLP) to healthy newborns before and after cheiloplasty. Additionally, a palatal growth curve is constructed to give more information about the natural growth before surgical intervention.

**Methods:**

Twenty-eight newborns with complete UCLP were enrolled in this study. Multiple plaster-casts of each child during their first year were collected and grouped in before and after cheiloplasty. A previous developed semi-automatic segmentation tool was used to assess the maxillary dimensions and were compared to a healthy control group. *Z*-scores were calculated to indicate differences between the two populations and if cheiloplasty had influence on maxillary growth. Furthermore, the prediction model created in a previous study was used to indicate differences between predictions and the outcome in UCLP measurements. The analysis was tested for inter- and intra-observer variability.

**Results:**

Results show differences in alveolar and palatal shape in UCLP patients in comparison with healthy controls. Prior to cheiloplasty an increased width and alveolar length was observed while the palatal depth was decreased. After cheiloplasty the widths moved towards normal but were still significantly larger.

**Conclusion:**

Infants with unilateral cleft lip and palate show a wider maxillary arch in comparison with the control population. Initial treatment has most influence on the width of the arch, which decreased towards normal.

## Introduction

Orofacial clefts are one of the most common congenital disorders with a prevalence in Europe about 0.9 cases per 1,000 livebirths (Calzolari et al., 2004). Children born with orofacial clefts often suffer from impaired functions like eating, speaking, and hearing. Additionally, the well-being of the child is often affected due to aesthetic and psychological problems (Shaw et al., 2001; Bos & Prahl, 2011). Unilateral cleft lip and palate (UCLP) is the most common type of orofacial cleft (Jagomagi, Soots & Saag, 2010; Rusková et al., 2014; Yilmaz, Ozbilen & Ustun, 2019).

It is known that corrective surgery for UCLP, e.g., lip closure (cheiloplasty) and palate closure (palatoplasty), has influence on normal maxillary growth (Kuijpers-Jagtman & Long, 2000). Multiple studies report that timing, surgical technique and expertise of the surgeon all may have impact on craniofacial development (Kuijpers-Jagtman & Long, 2000; Peltomäki et al., 2001).

Few studies have investigated the effect of cheiloplasty on the maxillary dental arch and palate. The findings are contradicting, Huang et al. (2002) investigated the effect of cheiloplasty at 3 months of age on the development of the maxillary arch and reported an inhibitory effect on the anterior maxillary arch width after surgery. A drawback of this study is that measurements were performed using a slide caliper and the post-cheiloplasty dataset was seven months after surgery and thus included the effect of normal growth as well. In contrast, a study of Kongprasert et al. (2019), who used the same surgical technique at a median of 4 months, did not show an effect on the maxillary arch width, but found that the dental arch bended palatally.

The Eurocleft project (Shaw et al., 2001) performed between 1996 and 2000 showed that there was almost no consensus about the best treatment protocol. They concluded that 195 protocols in 201 different cleft centers were present to treat newborns with UCLP. Protocols were mainly based on the team’s expertise and philosophy rather than a solid scientific base to see which treatment and technique performs best on the individual patient. After the project, a number of Scandinavian and British cleft centers started the Scandcleft project (Semb et al., 2017) that implied three randomized controlled trials for lip and palate closure tested against a common method. In their work they concluded that the influence on maxillary growth was primary affected by the surgeons’ familiarity with the surgical technique while the other surgical factors did not influence the outcome. (Rautio et al., 2017; Shaw & Semb, 2017).

For evaluation of treatment results plaster casts with use of calipers are still used, but digital three-dimensional (3D) casts are getting more accepted. Digital casts have several advantages over traditional casts in terms of availability (copies can be sent easily), storage (no need for physical storage) and safety (no breakage, and digital backups can easily be made) (Fleming, Marinho & Johal, 2011; Fernandes et al., 2015; Camardella et al., 2020). Although multiple studies have reported a small decrease of repeatability for linear measurements as compared to plaster models (Abizadeh et al., 2012; De Waard et al., 2014), digital models can be used for more complex applications like 3D area/volume calculations, superimposition with other models, digital manipulation and use of digital algorithms (Fleming, Marinho & Johal, 2011).

In our previous study (Bruggink et al., 2019), maxillary arch growth models were created to describe growth in young healthy infants. These models can be used to investigate if infants with UCLP express different maxillary arch growth. The aim of this study was to investigate the effect of cheiloplasty on maxillary arch dimensions. A secondary aim was to compare maxillary dimensions in infants with and without UCLP throughout the first 12 months after birth.

## Materials & Methods

### Subjects

Infants with non-syndromic UCLP born between 1972 and 1998 were included in this study. The patients were treated from birth on by the cleft palate craniofacial team of the Radboud University Medical Center, Nijmegen, The Netherlands. All infants were Caucasian, had no known syndrome and had no Simonart’s band present. Furthermore, a minimum of two casts in the first year were required to be able to determine growth.

Dental casts made at regular appointments during cleft care in the first year of life were collected. Casts were classified and grouped as taken before or after cheiloplasty. Cheiloplasty was performed according to Millard’s technique at four to eight months of age after presurgical orthopedic treatment with a passive maxillary plate.

The control population was derived from an interdisciplinary prospective growth and development study of Kramer et al. (Kramer, Hoeksma & Prahl-Andersen, 1989; Kramer, Hoeksma & Prahl-Andersen, 1992; Felix-Schollaart, Hoeksma & Prahl-Andersen, 1992) and contained 70 healthy infants without any congenital anomalies. These babies were all from Caucasian birth, were born full term and had no first- to third-degree relative with an oral cleft.

All data were anonymized prior to analysis. Ethical approval from the regional institutional review board was obtained for this study (Research Ethics Committee (CMO), Region Arnhem/Nijmegen, The Netherlands (2016-2654)). This study was conducted in compliance with the World Medical Association Declaration of Helsinki on medical research ethics.

A power analysis was performed using G*Power 3.1.9.7 (Faul et al., 2007) on data from an earlier study of Kramer, Hoeksma & Prahl-Andersen (1994). With a power of 80% and a significance of 5%, the minimal required sample size was 28 (independent groups and two-tailed analysis). The results of the power analysis can be seen in Appendix S3.

### Data acquisition

Maxillary plaster casts were digitized using the 3Shape R500 3D Dental Laser scanner (3Shape^®^, Copenhagen, Denmark). Scans were made using the high-resolution setting, producing a spatial resolution of 0.01 mm as specified by the manufacturer. The 3D digitized casts were checked for errors and exported to Standard Tessellation Language (STL) files.

### Data analysis

**Figure 1:**
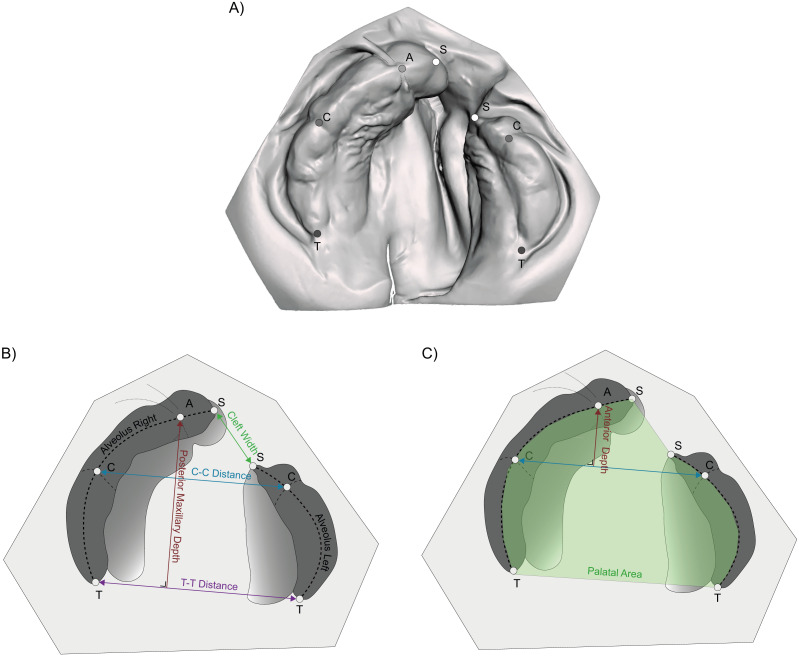
Location of the manual placed landmarks and the used parameters. (A) T, Tuberosity point; C, Cuspid point; A, Frontal point; and S, Segment point; (B) C-C distance, distance between both cuspids; T-T distance, distance between both tubers; Posterior Maxillary depth, the length of the perpendicular line between both tubers and the frontal point; (C) Anterior Maxillary depth, the length of the perpendicular line between both cuspids and the frontal point; Alveolar length, the combination of the lengths of the left and right calculated alveolus and the Palatal Area, 2D area enclosed by the alveolar arch.

Maxillary arch parameters used in our previous study were used to describe the maxillary arch (Bruggink et al., 2019). These were evaluated using a custom-made software program written in Matlab (MATLAB^®^ 2018b, The Mathworks, Inc., Natick, Massachusetts, USA). The parameters were based on five reproducible landmarks; the two tuberosities (T), the two cuspid points (C) and a point placed on the alveolar ridge between the superior labial frenulum and the papilla (A). Two landmarks were added to the anterior border of both alveolar segments to identify the cleft (S). The manual placed landmarks are shown in Fig. 1A.

The maxillary arch width was determined by the distance between both cuspid and tuberosity points, labelled respectively as the inter-cuspid (C-C) and inter-tuber (T-T) distance. The maxillary arch depth was divided in an anterior and total depth. These parts were calculated as the length of a perpendicular line between respectively the two cuspids and tuberosities with point A on the arch. The alveolar arch length resulted from a semi-automatic analysis determining the alveolar ridge in edentate plaster casts and was used as the border to calculate the palatal area. Finally, the cleft width is the distance between both segment identifiers (S-S) The parameters that are used, are visualized in Fig. 1B

To reconstruct control data corresponding with the time points of the UCLP plater clasts, the time points were inserted into the growth models describing the maxillary arch in control infants. Comparison of growth was performed with use of z-scores, indicating the amount of standard deviations the parameter in the UCLP population differs from the controls. *Z*-scores are often used in anthropometric growth studies to reflect the reference population. (Dibley et al., 1987) Furthermore, these growth models were used to create a growth model for each maxillary arch parameter in the UCLP population.

### Statistical analysis

Descriptive statistics were performed for all parameters. T-tests were performed to indicate differences between sexes. The UCLP casts were designated into a pre-cheiloplasty (T0) and post-cheiloplasty (T1) group. No grouping of the control group was needed as the corresponding parameters of the control group were calculated with the corresponding time points of the UCLP group.

The analysis consisted out of answering two main questions:

• What are the differences in maxillary arch dimensions and growth between the UCLP and the control group?
• What is the effect of cheiloplasty on the dimensions of the maxillary dental arch in the UCLP group?

Concerning the first question, all differences with the control population were calculated as *z*-scores because the maxillary arch dimensions have different scales and due to growth will change considerably by increasing age. This allowed for easy comparison both between dimensions as well as between different points in time.

For the second question, only the casts closest prior to and after cheiloplasty were included. Patients lacking one of these casts were excluded. Paired-sample t-tests were performed to test if the cheiloplasty had influence on the maxillary arch dimensions expressed in mm.

Growth models based on Linear Mixed Models were created for the parameters in the UCLP population as done in the previous study (Bruggink et al., 2019). These were plotted against the existing models present for the control group for visualization purposes. As the number of degrees in the polynomial functions describing growth model did not always correspond with each other, these could not be used for statistical comparison.

### Reliability of the method

Two observers performed repeated measurements of 20 randomly chosen casts derived from the ULCP group. The intra- and inter-observer variability was tested using the Pearson correlation. Systematic differences were assessed with paired-sample t-tests. To indicate the random error the Duplicate Measurement Error (DME) was calculated, which is calculated by dividing the standard deviation by $\sqrt{2}$. For all tests the significance level was set at *p* < 0.05. The reliability of the control population was performed in an earlier study and showed a high inter- and intra-observer agreement. (Bruggink et al., 2019)

## Results

### Reliability of the method

The results of the inter- and intra-observer reliability tests are shown in Table 1. The correlation within observers was between 0.88 and 0.97 and the two-sampled *t*-test showed that none of the parameters were significant different. Between observers a correlation of 0.79 and 0.92 was observed. The total maxillary depth (0.82 mm), alveolar length (2.47 mm) and palatal area (38.80 mm^2^) differed significantly between observers.

**Table 1:** Intra- and interobserver reliability. The random error is shown by the duplicate measurement error (DME). The mean difference tested with the paired sample *t*-test indicates the systematic differences.

**Parameter**	**Correlation coefficient**	**DME**	**Mean difference**	**95% CI**	**p**
**Intra-observer**					
TT distance (mm)	0.97	0.50	0.16	[−0.18 to 0.50]	0.34
CC distance (mm)	0.93	0.73	−0.11	[−0.61 to 0.38]	0.64
Anterior maxillary depth (mm)	0.94	0.43	−0.24	[−0.53 to 0.05]	0.10
Maxillary depth (mm)	0.96	0.54	−0.19	[−0.56 to 0.17]	0.28
Alveolar length (mm)	0.84	3.05	−0.95	[−3.03 to 1.13]	0.35
Cleft width (mm)	0.92	0.91	−0.06	[−0.68 to 0.56]	0.84
Palatal area (mm^2^)	0.88	38.80	−7.78	[−34.22 to 28.67]	0.54
**Inter-observer**					
TT distance (mm)	0.90	0.87	−0.49	[−1.09 to 0.10]	0.10
CC distance (mm)	0.81	1.24	0.71	[−0.13 to 1.56]	0.09
Anterior maxillary depth (mm)	0.79	0.81	−0.38	[−0.94 to 0.16]	0.16
Maxillary depth (mm)	0.91	0.84	0.82	[0.25 to 1.40]	<0.01
Alveolar length (mm)	0.84	1.66	2.47	[1.34 to 3.60]	<0.01
Cleft width (mm)	0.92	0.78	−0.24	[0.78 to 0.29]	0.35
Palatal area (mm^2^)	0.88	41.13	41.26	[13.23 to 69.30]	<0.01

### Sample descriptive

A database search resulted in 139 recorded patients for whom it is was known that plaster casts were taken. After visual inspection of the models, only 40 patients had two or more models in their first year. Eight patients were excluded as the surgery date was unknown and four were excluded due to a diagnosed syndrome. This resulted in a total of 28 included patients. After infant orthopedic treatment the lip was closed at a mean age of 209 ± 48 days. Initially 160 casts were collected and scanned. According to the date of cheiloplasty 124 casts were grouped in T0 and 36 in T1. Only a significant sex difference was found for the anterior arch depth; The results of this analysis can be found in Appendix S1. An additional three patients were excluded for comparing the effect of cheiloplasty as they missed a pre- or post-cheiloplasty cast. However, these data can still be used in the growth models. The mean difference between the pre- and post-cheiloplasty casts was 74 ± 32 days.

### Maxillary arch analysis

The inter-tuber distance, inter-cuspid distance, anterior maxillary arch depth and the alveolar length were significantly larger in the UCLP group while the total maxillary arch depth was lower than those based on the healthy controls, both in the pre- and post-cheiloplasty periods (Table 2). After cheiloplasty, the anterior maxillary arch depth plus the inter-cuspid and inter-tuber distances shifted more towards the control population. For the anterior maxillary arch depth, no significant difference with the healthy population was present. In contradiction, the length of the alveolar arch moved away from the controls by growing more quickly than in the UCLP group.

**Table 2:** Differences in maxillary dimensions between UCLP patients and the growth model for healthy controls, expressed as *z*-scores. The results are grouped before and after cheiloplasty.

	**Before Cheiloplasty**			**After Cheiloplasty**		
**Parameter**	**Mean (SD)**	**25**th**–75**th **percentile**	**p**	**Mean (SD)**	**25**th **–75**th **percentile**	**p**
TT distance (z)	3.82 (1.70)	2.77–5.06	<0.01	2.95 (1.62)	1.65–4.11	<0.01
CC distance (z)	3.67 (1.84)	2.24–4.89	<0.01	2.75 (1.57)	1.74–4.34	<0.01
Anterior maxillary depth (z)	1.32 (1.83)	0.17–2.51	<0.01	0.03 (2.17)	−1.08–0.99	0.77
Maxillary depth (z)	−1.00 (1.45)	−2.10–0.00	<0.01	−0.97 (1.74)	−2.23–0.16	<0.01
Alveolar length (z)	1.24 (2.04)	−0.14–2.75	<0.01	2.72 (1.54)	1.57–4.13	<0.01

The effect of cheiloplasty on the parameters based only on the UCLP population is shown in Table 3. A significant decrease of the inter-cuspid distance (−0.70 ± 1.35 mm), cleft width (−2.52 ± 2.83 mm) and anterior maxillary depth (−0.68 ± 1.41 mm) was observed.

**Table 3:** The influence of cheiloplasty on the dimensions of the maxilla expressed as the difference in millimeters.

**Parameter**	**Mean just before cheiloplasty (SD)**	**Mean just after cheiloplasty (SD)**	**Mean difference (SD)**	**95% CI of difference**	*p*
TT distance (mm)	35.16 (3.18)	35.41 (3.60)	0.24 (1.77)	[−0.51 to 0.99]	0.51
CC distance (mm)	34.23 (3.46)	33.53 (3.15)	−0.70 (1.35)	[−1.26 to −0.12]	0.02
Anterior maxillary depth (mm)	9.69 (1.65)	9.01 (1.69)	−0.68 (1.41)	[−1.26 to −0.10]	0.02
Maxillary depth (mm)	28.71 (2.11)	28.72 (2.63)	0.01 (2.27)	[−0.95 to 0.97]	0.98
Alveolar length (mm)	85.00 (6.12)	87.10 (6.37)	2.10 (5.87)	[−0.37 to 4.58]	0.09
Cleft Width (mm)	8.84 (4.75)	6.33 (3.46)	−2.52 (2.83)	[−3.70 to −1.35]	<0.01
2D palatal area (mm^2^)	913.00 (86.12)	911.94 (81.24)	−1.06 (69.98)	[−30.61 to 28.49]	0.94

**Figure 2:**
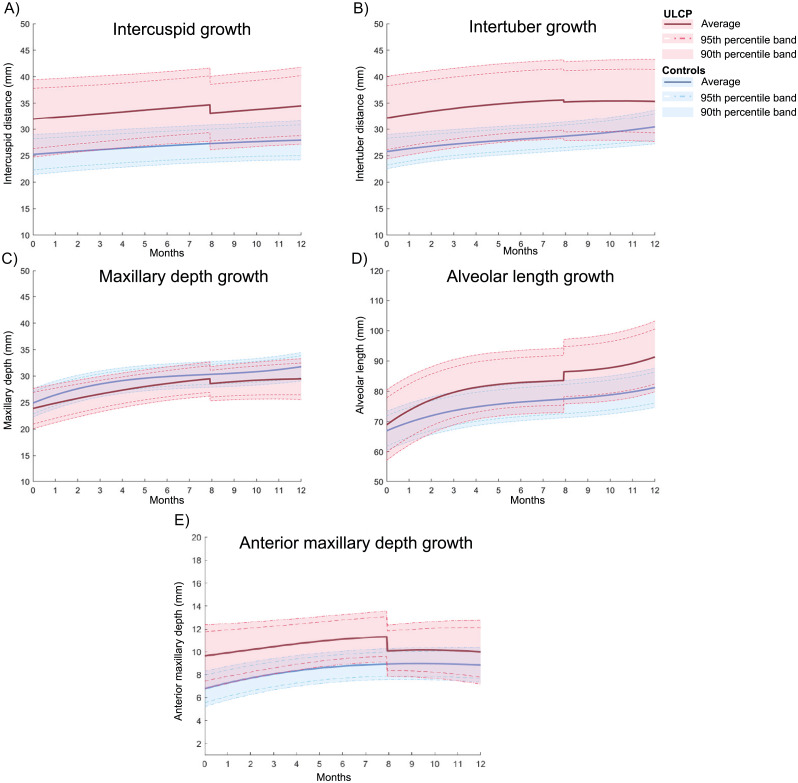
UCLP Growth curves compared with healthy controls within the first year of birth. The curves are drawn with their average growth and the 90 and 95% interval. The sharp twist at around 8 months is the average time of cheiloplasty which is chosen to differentiate between pre- and post-surgery. The growth curves are shown for the C-Cdistance (A), T-T distance (B), maxillary depth (C), alveolar length (D) and the anterior maxillary depth (E).

In Fig. 2 the growth casts of both UCLP and the control population are shown in their first year after birth. At an average of eight months cheiloplasty took place and causes an offset in the ULCP curves. The parameters describing these curves are listed in Appendix S2

## Discussion

Infants born with UCLP are exposed to interventions like infant orthopedics and cheiloplasty at an early stage of their lives. This study examined, with use of digital 3D casts, if this population expressed different maxillary dimensions in comparison with non-cleft healthy born children. Additionally, the direct effect of cheiloplasty on these dimensions was investigated.

The manual placement of landmarks on 3D casts used in this study can introduce unreliability. It is of major importance to quantify these errors before analyzing the data. As shown in Table 1, agreement between observers in this study was high, which is in correspondence with our previous study (Bruggink et al., 2019). However, there was a significant mean difference of about 2.47 mm for the alveolar length and 41.26 mm^2^ for the palatal area between observers. Within observers these values were far less, but with a high random error (DME) as well. In addition to our previous study, the alveolar arch was determined with two extra landmarks to account for the cleft. However, the exact location of the alveolar interruption was often not clear. In many cases the alveolus bends slightly into the cleft area, making it difficult to identify the border of the alveolar segment. In this study it was tried to overcome this by using the most medial point on the alveolus from a caudal view. However, it turned out to be still difficult to determine these points reliably. Areal comparisons with healthy controls were excluded as the borders of the cleft add a lot of uncertainty. Accurate determination of the cleft’s boundaries is difficult as the border between the hard palate and nasal tissues is not clear in most cases. This was often caused by the quality of the casts and due to the use of gauze pads to cover the cleft during impression taking, preventing the acquisitions of undercuts at the border. However, the change in two-dimensional area of the palatal area can give additional information about the effect of an intervention on the size of e.g., the palate or cleft.

As seen from the control data of healthy infants in Fig. 2, the parameters determining the width and depth of the palate were almost identical for all infants, resulting in a round shape of the maxillary arch. The UCLP population showed significant wider maxillary arch widths (inter-tuber distance) in comparison to the values derived from the growth model for the healthy population. In addition, with the observed smaller maxillary arch depth, it can be indicated that a shift towards an ellipsoid shape in the UCLP group was present. After cheiloplasty, the z-scores of the anterior width decreased towards the control population. This can be explained by the increase of lip pressure occurring after lip closure. A study of Bardach et al. (1984) showed that lip pressure is significantly increased after cheiloplasty and that this pressure remains higher during their follow-up period of two years, acting as an important modulating factor for craniofacial development. This phenomenon was supported by the decrease of the anterior maxillary arch depth, while no change in the total depth was observed, indicating that the effects are most present anteriorly. Despite the decrease of the width, the dimensions were still significant larger in comparison with the controls. According to a study of Reiser, Skoog & Andlin-Sobocki (2013) the maxillary arch width remains larger until the closure of the hard palate, after which the transversal growth is inhibited.

Investigating only the effect of cheiloplasty in the UCLP group, the anterior maxillary arch width was observed to diminish, directing towards normal values (Table 3). This finding was supported by other studies (Honda et al., 1995; Kramer, Hoeksma & Prahl-Andersen, 1996; Prahl et al., 2001; Huang et al., 2002; Falzoni et al., 2016; Ambrosio et al., 2018). However, a recent study of Hoffmannova et al. (2018) showed an increase instead, which they explained as the effect of a different surgical technique. They used a modified Tennison’s technique instead of the Millard’s technique used in the present study. The modified Tennison’s technique is assumed to exert less lip pressure and would therefore cause less effect on the maxillary arch. Another possible explanation is that this study had a large time interval of ten months between the pre- and post- cheiloplasty cast, introducing -besides the effect of cheiloplasty - maxillary growth as well.

Although the posterior width did move towards the control population after cheiloplasty, the direct effect of the cheiloplasty showed a small non-significant increase in the inter-tuber distance. This can be explained by the increased inter-tuber growth present in the control population around the timing of cheiloplasty (Fig. 2). Furthermore, it could be possible that the increased lip pressure causes rotation of the maxillary segments at the sphenoid region causing the anterior part to move medially, while the posterior part rotate more laterally. Although the increase of the intertuber distance was in our study not statistically significant, other studies do corroborate this as in most cases the inter-tuber distance stabilized or increased after cheiloplasty (Kramer, Hoeksma & Prahl-Andersen, 1994; Honda et al., 1995; Huang et al., 2002; Falzoni et al., 2016; Ambrosio et al., 2018; Hoffmannova et al., 2018). This effect can also have caused the decrease of the cleft width which is reported in other studies as well (Huang et al., 2002; Eichhorn et al., 2011).

However, it must be stated that the DME values were larger than the differences found before and after cheiloplasty for most parameters. This can indicate that a part of these differences could be related to intra-observer variability.

One of the limits of this study is the differences in cast acquisition between the control and UCLP group. The casts in the control group were made following a strict research protocol, acquiring a cast every 3 months after birth in the first year. The casts for the UCLP population were made during regular appointments, which did not correspond with the timestamps from the controls. Therefore, modelling and using Z-scores was needed to compare the groups, introducing some degree of uncertainty. Furthermore, as stated in the methods section, it was not possible to directly compare the growth models between both populations, as the amount of describing coefficients were not the same for each parameter. The created growth curves are shown in Appendix S2. and visualized in Fig. 2. For the calculations of the growth models all 3D casts were included, instead of only the first prior and after cheiloplasty. This can induce small discrepancies between this figure and Table 3.

To summarize, the results showed altered dimensions in the UCLP population, the maxillary arch shape is more ellipsoid due to a wide maxillary arch. After cheiloplasty the transversal parameters tended to normalize but were still bigger than in the controls. At last, cheiloplasty had mainly an effect on the anterior part of the palatal arch.

With the present study, we examined the effect of cheiloplasty on the maxillary arch in children born with UCLP. With this information a better rationale can be made to choose for the best patient specific protocol. It is also important to investigate the growth differences at a later stage to examine the effect of e.g., palatoplasty and alveolar bone grafting as these could distort the growth as well (Hoffmannova et al., 2016). When this information can be used to minimize or prevent distortion of healthy maxillary growth, the number of further reconstructive procedures could be decreased. The current algorithm to determine the alveolar arch does not work with dentate casts, and therefore should be updated to be more robust to include casts over a year.

## Conclusions

In unilateral cleft lip and palate, a wider maxillary arch is observed in comparison with the control population. Initial treatment consisting of infant orthopedics plus cheiloplasty influences maxillary arch dimensions. This is most visible on the transversal parameters, which decreased towards the normal population. The models for normal maxillary arch growth also create opportunities for comparing to other craniofacial abnormalities.

##  Supplemental Information

10.7717/peerj.9631/supp-1 Supplemental Information 1 The differences between genders included in this study

10.7717/peerj.9631/supp-2 Supplemental Information 2 The resulting properties for the statistical random effect growth model

10.7717/peerj.9631/supp-3 Supplemental Information 3 Results of the power analysis These data derived from the study of Kramer et al. (Kramer, Hoeksma & Prahl-Andersen, 1994). The anterior depth at 6 months is ignored as this one is too high.

10.7717/peerj.9631/supp-4 Supplemental Information 4 Raw SPSS data: The results of our analysis

10.7717/peerj.9631/supp-5 Supplemental Information 5 Matlab scripts for landmarking and analysis

## Additional Information and Declarations

### Data Availability

The following information was supplied regarding data availability: Raw data and Matlab scripts are available in the Supplementary Files.

## References

[ref-1] Abizadeh N, Moles DR, O’Neill J, Noar JH (2012). Digital versus plaster study models: how accurate and reproducible are they?. Journal of Orthodontics.

[ref-2] Ambrosio ECP, Sforza C, De Menezes M, Gibelli D, Codari M, Carrara CFC, Machado MAAM, Oliveira TM (2018). Longitudinal morphometric analysis of dental arch of children with cleft lip and palate: 3D stereophotogrammetry study. Oral Surgery, Oral Medicine, Oral Pathology and Oral Radiology.

[ref-3] Bardach J, Bakowska J, McDermott-Murray J, Mooney MP, Dusdieker LB (1984). Lip pressure changes following lip repair in infants with unilateral clefts of the lip and palate. Plastic and Reconstructive Surgery.

[ref-4] Bos A, Prahl C (2011). Oral health-related quality of life in Dutch children with cleft lip and/or palate. The Angle Orthodontist.

[ref-5] Bruggink R, Baan F, Kramer GJC, Maal TJJ, Kuijpers-Jagtman AM, Bergé SJ, Bronkhorst EM, Ongkosuwito EM (2019). Three dimensional maxillary growth modeling in newborns. Clinical Oral Investigations.

[ref-6] Calzolari E, Bianchi F, Rubini M, Ritvanen A, Neville AJ, Dolk H (2004). Epidemiology of cleft palate in Europe: implications for genetic research. Cleft Palate-Craniofacial Journal.

[ref-7] Camardella LT, Ongkosuwito EM, Penning EW, Kuijpers-Jagtman AM, Vilella OV, Breuning KH (2020). Accuracy and reliability of measurements performed using two different software programs on digital models generated using laser and computed tomography plaster model scanners. Korean Journal of Orthodontics.

[ref-8] De Waard O, Rangel FA, Fudalej PS, Bronkhorst EM, Kuijpers-Jagtman AM, Breuning KH (2014). Reproducibility and accuracy of linear measurements on dental models derived from cone-beam computed tomography compared with digital dental casts. American Journal of Orthodontics and Dentofacial Orthopedics.

[ref-9] Dibley MJ, Staehling N, Nieburg P, Trowbridge FL (1987). Interpretation of Z-score anthropometric indicators derived from the international growth reference. American Journal of Clinical Nutrition.

[ref-10] Eichhorn W, Blessmann M, Vorwig O, Gehrke G, Schmelzle R, Heiland M (2011). Influence of lip closure on alveolar cleft width in patients with cleft lip and palate. Head and Face Medicine.

[ref-11] Falzoni M, Jorge P, Laskos K, Carrara C, Machado M, Valarelli F, Oliveira T (2016). Three-dimensional dental arch evaluation of children with unilateral complete cleft lip and palate. Dental, Oral and Craniofacial Research.

[ref-12] Faul F, Erdfelder E, Lang AG, Buchner A (2007). G*Power 3: a flexible statistical power analysis program for the social, behavioral, and biomedical sciences. Behavior Research Methods.

[ref-13] Felix-Schollaart B, Hoeksma JB, Prahl-Andersen B (1992). Growth comparison between children with cleft lip and/or palate and controls. Cleft Palate-Craniofacial Journal.

[ref-14] Fernandes VM, Jorge PK, Carrara CFC, Gomide MR, Machado MAAMMAAM, Oliveira TM (2015). Three-dimensional digital evaluation of dental arches in infants with cleft lip and/or palate. Brazilian Dental Journal.

[ref-15] Fleming PS, Marinho V, Johal A (2011). Orthodontic measurements on digital study models compared with plaster models: a systematic review. Orthodontics and Craniofacial Research.

[ref-16] Hoffmannova E, Moslerová V, Dupej J, Borský J, Bejdová, Velemínská J (2018). Three-dimensional development of the upper dental arch in unilateral cleft lip and palate patients after early neonatal cheiloplasty. International Journal of Pediatric Otorhinolaryngology.

[ref-17] Hoffmannova E, Š Bejdová, Borský J, Dupej J, Cagáňová V, Velemínská J (2016). Palatal growth in complete unilateral cleft lip and palate patients following neonatal cheiloplasty: classic and geometric morphometric assessment. International Journal of Pediatric Otorhinolaryngology.

[ref-18] Honda Y, Suzuki A, Ohishi M, Tashiro H (1995). Longitudinal study on the changes of maxillary arch dimensions in japanese children with cleft lip and/or palate: infancy to 4 years of age. The Cleft Palate-Craniofacial Journal.

[ref-19] Huang C-S, Wang W-I, Liou EJ-W, Chen Y-R, Chen PK-T, Noordhoff MS (2002). Effects of cheiloplasty on maxillary dental arch development in infants with unilateral complete cleft lip and palate. The Cleft Palate-Craniofacial Journal.

[ref-20] Jagomagi T, Soots M, Saag M (2010). Epidemiologic factors causing cleft lip and palate and their regularities of occurrence in Estonia. Stomatologija/issued by public institution Odontologijos studija.

[ref-21] Kongprasert T, Winaikosol K, Pisek A, Manosudprasit A, Manosudprasit A, Wangsrimongkol B, Pisek P (2019). Evaluation of the effects of cheiloplasty on maxillary arch in UCLP infants using three-dimensional digital models. Cleft Palate-Craniofacial Journal.

[ref-22] Kramer GJC, Hoeksma JB, Prahl-Andersen B (1989). Emergence of the deciduous incisors in CLP children. European Journal of Orthodontics.

[ref-23] Kramer GJC, Hoeksma JB, Prahl-Andersen B (1992). Early palatal changes in complete and incomplete cleft lip and/or palate. Cells Tissues Organs.

[ref-24] Kramer GJC, Hoeksma JB, Prahl-Andersen B (1994). Palatal changes after lip surgery in different types of cleft lip and palate. Cleft Palate-Craniofacial Journal.

[ref-25] Kramer GUC, Hoeksma JB, Prahl-Andersen B (1996). Early palatal changes after initial palatal surgery in children with cleft lip and palate. Cleft Palate-Craniofacial Journal.

[ref-26] Kuijpers-Jagtman AM, Long J (2000). The influence of surgery and orthopedic treatment on maxillofacial growth and maxillary arch development in patients treated for orofacial clefts. Cleft Palate-Craniofacial Journal.

[ref-27] Peltomäki T, Vendittelli BL, Grayson BH, Cutting CB, Brecht LE (2001). Associations between severity of clefting and maxillary growth in patients with unilateral cleft lip and palate treated with infant orthopedics. Cleft Palate-Craniofacial Journal.

[ref-28] Prahl C, Kuijpers-Jagtman AM, Van’T Hof MA, Prahl-Andersen B (2001). A randomised prospective clinical trial into the effect of infant orthopaedics on maxillary arch dimensions in unilateral cleft lip and palate (Dutchcleft). European Journal of Oral Sciences.

[ref-29] Rautio J, Andersen M, Bolund S, Hukki J, Vindenes H, Davenport P, Arctander K, Larson O, Berggren A, Åbyholm F, Whitby D, Leonard A, Lilja J, Neovius E, Elander A, Heliövaara A, Eyres P, Semb G (2017). Scandcleft randomised trials of primary surgery for unilateral cleft lip and palate: 2. Surgical results. Journal of Plastic Surgery and Hand Surgery.

[ref-30] Reiser E, Skoog V, Andlin-Sobocki A (2013). Early dimensional changes in maxillary cleft size and arch dimensions of children with cleft lip and palate and cleft palate. Cleft Palate-Craniofacial Journal.

[ref-31] Rusková H, Bejdová Š, Peterka M, Krajíček V, Velemínská J (2014). 3-D shape analysis of palatal surface in patients with unilateral complete cleft lip and palate. Journal of Cranio-Maxillofacial Surgery.

[ref-32] Semb G, Enemark H, Friede H, Paulin G, Lilja J, Rautio J, Andersen M, Åbyholm F, Lohmander A, Shaw W, Mølsted K, Heliövaara A, Bolund S, Hukki J, Vindenes H, Davenport P, Arctander K, Larson O, Berggren A, Whitby D, Leonard A, Neovius E, Elander A, Willadsen E, Bannister RP, Bradbury E, Henningsson G, Persson C, Eyres P, Emborg B, Kisling-Møller M, Küseler A, Black BGranhof, Schöps A, Bau A, Boers M, Andersen HS, Jeppesen K, Marxen D, Paaso M, Hölttä E, Alaluusua S, Turunen L, Humerinta K, Elfving-Little U, Tørdal IB, Kjøll L, Aukner R, Hide Ø, Feragen KB, Rønning E, Skaare P, Brinck E, Semmingsen AM, Lindberg N, Bowden M, Davies J, Mooney J, Bellardie H, Schofield N, Nyberg J, Lundberg M, Karsten ALA, Larson M, Holmefjord A, Reisæter S, Pedersen NH, Rasmussen T, Tindlund R, Sæle P, Blomhoff R, Jacobsen G, Havstam C, Rizell S, Enocson L, Hagberg C, Najar Chalien M, Paganini A, Lundeborg I, Marcusson A, Mjönes AB, Gustavsson A, Hayden C, McAleer E, Slevan E, Gregg T, Worthington H (2017). A Scandcleft randomised trials of primary surgery for unilateral cleft lip and palate: 1. Planning and management. Journal of Plastic Surgery and Hand Surgery.

[ref-33] Shaw W, Semb G (2017). The Scandcleft randomised trials of primary surgery for unilateral cleft lip and palate: 11. What next?. Journal of Plastic Surgery and Hand Surgery.

[ref-34] Shaw WC, Semb G, Nelson P, Brattström V, Mølsted K, Prahl-Andersen B, Gundlach KKH (2001). The Eurocleft project 1996–2000: overview. Journal of Cranio-Maxillofacial Surgery.

[ref-35] Yilmaz HN, Ozbilen EO, Ustun T (2019). The prevalence of cleft lip and palate patients: a single-center experience for 17 years. Turkish Journal of Orthodontics.

