# Supplementary material for: The effect of lip closure on palatal growth in patients with unilateral clefts
Source: PeerJ. 2020 Jul 30;8:e9631. doi: 10.7717/peerj.9631 (PMC7396139; doi:10.7717/peerj.9631)
Supplement: Supplemental Information 1 [file peerj-08-9631-s001.docx]

# Appendix 1, Differences between genders

| **Parameter** | **Mean Difference (SD)** | **95% CI of difference** | **p** |
| --- | --- | --- | --- |
| TT Distance (mm) | 0.67 (0.52) | [-0.35 to 1.70] | 0.20 |
| CC Distance (mm) | -0.02 (0.58) | [-1.17 to 1.13] | 0.97 |
| Maxillary Depth (mm) | -0.45 (0.47) | [-1.38 to 0.48] | 0.34 |
| Anterior Maxillary Depth (mm) | -0.75 (0.27) | [-1.29 to -0.21] | <0.01 |
| Cleft width | -0.24 (0.71) | [-1.65 to 1.17] | 0.74 |
| Alveolar Length (mm) | -0.40 (1.49) | [-3.34 to 2.54] | 0.79 |
| Palatal Area (mm^2^) | 0.94 (19.23) | [-37.04 to 38.93] | 0.96 |

Appendix 1 - The differences between genders included into this study
