# Supplementary material for: The effect of lip closure on palatal growth in patients with unilateral clefts
Source: PeerJ. 2020 Jul 30;8:e9631. doi: 10.7717/peerj.9631 (PMC7396139; doi:10.7717/peerj.9631)
Supplement: Supplemental Information 2 [file peerj-08-9631-s002.docx]

# Appendix 2, ULCP linear growth model parameters

|  |  | **Fixed Effect (size / se; p)** | | | | **Random Effect (sd)** | | | |
| --- | --- | --- | --- | --- | --- | --- | --- | --- | --- |
| **Parameter** | **Intercept** | **Time** | **Time^2^** | **Time^3^** | **Closure Effect** | **Intercept** | **Time** | **Time^2^** | **Residual** |
| *CC (mm)* | 32.48 | 4.04 | n.a. | n.a. | 1.60 | 3.48 | 3.34 | n.a. | 0.99 |
|  | 0.69; <0.001 | 0.74; <0.001 | n.a. | n.a. | 0.31; 0.013 |  | | | |
| *TT (mm)* | 34.81 | 3.49 | -4.93 | n.a. | 0.38 | 3.15 | 2.48 | n.a. | 0.99 |
|  | 0.68; <0.001 | 0.82; <0.001 | 1.52; 0.983 | n.a. | 0.37; 0.156 |  | | | |
| *Alveolar Length (mm)* | 85.79 | 5.25 | -16.92 | 57.46 | -2.87 | 6.32 | 6.01 | n.a. | 3.54 |
|  | 1.63; <0.001 | 3.63; 0.075 | 6.18; 0.003 | 18.90; 0.001 | 1.29; <0.001 |  | | | |
| *Anterior Maxillary Depth (mm)* | 9.82 | 2.28 | -2.90 | n.a. | -0.98 | 1.34 | 2.02 | n.a. | 0.83 |
|  | 0.29; <0.001 | 0.64; <0.001 | 1.20; 0.017 | n.a. | 0.30; 0.001 |  | | | |
| *Maxillary Depth (mm)* | 27.70 | 6.50 | -5.78 | n.a. | 0.91 | 1.93 | 2.59 | n.a. | 1.29 |
|  | 0.55; <0.001 | 0.99; <0.001 | 1.96; 0.002 | n.a. | 0.48; 0.030 |  | | | |

*Appendix 2: The resulting properties for the statistical random effect growth model .*
