# Supplementary material for: The effect of lip closure on palatal growth in patients with unilateral clefts
Source: PeerJ. 2020 Jul 30;8:e9631. doi: 10.7717/peerj.9631 (PMC7396139; doi:10.7717/peerj.9631)
Supplement: Supplemental Information 3 — These data derived from the study of Kramer et al. (Kramer, Hoeksma & Prahl-Andersen, 1994). The anterior depth at 6 months is ignored as this one is too high. [file peerj-08-9631-s003.docx]

# Appendix 3, Results of the power analysis

| **Parameter** | **Anterior Depth (mm)** | | | **Total Depth (mm)** | | | **Inter-cuspid Distance (mm)** | | | **Inter-tuber Distance (mm)** | | |
| --- | --- | --- | --- | --- | --- | --- | --- | --- | --- | --- | --- | --- |
| **Month** | 3 | 6 | 9 | 3 | 6 | 9 | 3 | 6 | 9 | 3 | 6 | 9 |
| **Mean Control** | 8.5 | 8.6 | 9.3 | 28.7 | 29.1 | 30.8 | 26 | 26.7 | 27.1 | 27.3 | 27.9 | 28.9 |
| **Std Control** | 0.8 | 0.9 | 1 | 1.6 | 1.8 | 2.4 | 2 | 1.9 | 1.8 | 2 | 2.2 | 2.1 |
| **Mean ULCP** | 9.3 | 8.5 | 8.4 | 26.8 | 27.2 | 28.2 | 30.1 | 29.6 | 29.3 | 31.8 | 31.7 | 32 |
| **Std ULCP** | 1.2 | 1.3 | 1.3 | 2.2 | 2.3 | 2.3 | 3 | 2.8 | 2.7 | 2.5 | 2.2 | 2.7 |
| **Power** | 0.8 | 0.8 | 0.8 | 0.8 | 0.8 | 0.8 | 0.8 | 0.8 | 0.8 | 0.8 | 0.8 | 0.8 |
| **Significance** | 0.05 | 0.05 | 0.05 | 0.05 | 0.05 | 0.05 | 0.05 | 0.05 | 0.05 | 0.05 | 0.05 | 0.05 |
| **Sample Size** | 27 | 2113 | 28 | 18 | 20 | 14 | 8 | 12 | 19 | 6 | 7 | 6 |

Appendix 3 – The results of the power analysis. These data derived from the study of Kramer et al. (Kramer, Hoeksma & Prahl-Andersen, 1994). The anterior depth at 6 months is ignored as this one is too high.
